# Limited Clinical Benefit of Immune Checkpoint Inhibition in Ovarian Cancer with Opportunities in Selected Subtypes

**DOI:** 10.3390/ijms27114923

**Published:** 2026-05-29

**Authors:** Zuzanna Ratka, Andrzej Gamian, Marta Woźniak

**Affiliations:** 1 Department of Clinical and Experimental Pathology, Wroclaw Medical University, Marcinkowskiego 1, 50-368 Wroclaw, Poland; zuzanna.ratka@student.umw.edu.pl; 2 Hirszfeld Institute of Immunology and Experimental Therapy, Polish Academy of Sciences, 12 Rudolfa Weigla Str., 53-114 Wroclaw, Poland

**Keywords:** ovarian cancer, immune checkpoint inhibitors, immunotherapy, PD-1/PD-L1, CTLA-4, TIM-3, clear cell ovarian carcinoma, clinical trials

## Abstract

Epithelial ovarian cancer (EOC) remains one of the most lethal gynecologic malignancies, largely owing to advanced-stage presentation, high rates of relapse, and the eventual emergence of therapeutic resistance. Despite the transformative success of immune checkpoint inhibitors (ICIs) across multiple solid tumors, their clinical impact in ovarian cancer has been comparatively modest. This literature review provides a comprehensive synthesis of recent advances in ICI strategies for ovarian cancer (OC), with particular emphasis on phase II and III clinical trials evaluating programmed cell death protein 1 (PD-1), programmed death-ligand 1 (PD-L1), cytotoxic T-lymphocyte–associated protein 4 (CTLA-4), and T cell immunoglobulin and mucin-domain-containing-3 (TIM-3)-directed therapies. Accumulating evidence indicates that PD-1/PD-L1 monotherapy yields limited clinical activity in unselected OC populations, with low objective response rates and minimal survival benefit. Dual checkpoint blockade with PD-1 and CTLA-4 inhibitors demonstrates enhanced antitumor activity, particularly in clear cell ovarian carcinoma (CCOC), albeit at the expense of increased immune-related toxicity. Large randomized trials incorporating ICI into first-line chemotherapy or maintenance settings have largely failed to improve outcomes in biomarker-unselected cohorts. Available evidence demonstrates that combinatorial approaches integrating ICI with anti-angiogenic agents, PARP inhibitors, or neoadjuvant chemotherapy provide modest benefit in selected molecular and histologic subgroups. Early-phase investigations of TIM-3–targeting strategies further expand the immunotherapeutic landscape, although clinical efficacy remains preliminary. Current evidence underscores that OC is not uniformly responsive to immunotherapy and that rational combination strategies, biomarker-driven patient selection, and improved understanding of tumor immune microenvironment heterogeneity are essential to unlocking the full therapeutic potential of ICI in this disease.

## 1. Introduction

OC remains one of the most formidable and challenging entities in contemporary gynecologic oncology, distinguished by persistently high mortality rates and an insidious, frequently asymptomatic course during its initial phases of progression. According to GLOBOCAN 2022 estimates, more than 324,000 new cases and nearly 207,000 deaths were recorded worldwide, positioning OC as one of the leading causes of cancer-related mortality among women globally [1]. The median age at diagnosis is approximately 63 years, and the disease manifests far more frequently in older women than in younger cohorts. Moreover, both the incidence and mortality associated with OC are notably higher in developed regions compared with developing nations [2]. The stage at diagnosis of OC varies widely. High-grade serous carcinomas (HGSCs) are typically detected at advanced stages, frequently stage III or IV in FIGO classification, and represent the most aggressive subtype. By contrast, many non-serous tumors are found earlier. Consequently, five-year survival outcomes differ markedly, with the serous subtype showing substantially poorer prognoses compared with endometrioid, mucinous, and clear-cell variants [3]. There are many risk factors associated with higher OC prevalence. Mutations in BRCA1 and BRCA2 substantially elevate the risk of OC, conferring lifetime probabilities of roughly 39–44% and 11–17%, respectively [4]. Additional susceptibility genes, including KRAS, RAD51C, and TP53, are likewise implicated in the molecular pathogenesis of the disease [5]. Additionally, elevated risk has been associated with reproductive and hormonal factors (early menarche, late menopause, nulliparity, infertility, and especially estrogen-only HRT), lifestyle determinants such as central obesity, smoking, and high dietary fat intake, as well as emerging evidence implicating chronic psychological stress as an additional contributory factor [2,6,7].

OC does not represent a single, uniform disease but rather encompasses a heterogeneous group of tumors with distinct molecular, histologic, and clinical profiles. More than 90% are EOC, within which several biologically divergent subtypes are recognized. HGSC, comprising the majority of cases, is characterized by pervasive TP53-driven genomic instability [8]. CCOC, often arising in the context of endometriosis, displays marked resistance to platinum-based therapies, whereas endometrioid tumors, more common in younger women, frequently harbor ARID1A and PTEN mutations [9,10]. Mucinous carcinoma represents a rare entity with a unique biological signature, and low-grade serous carcinoma (LGSC) shows an indolent yet chemotherapy-refractory course [11]. Other cases include transitional cell (Brenner tumors), mixed, and undifferentiated subtypes. This pronounced heterogeneity underlies substantial variation in treatment responses and clinical outcomes, posing a major challenge to the development of broadly effective therapeutic strategies.

Clonal heterogeneity, which is reflected in the coexistence of multiple tumor subclones with distinct mutational and transcriptional profiles across different metastatic sites, disease stages, and microenvironmental contexts, constitutes a defining feature of OC biology. As shown by single-cell sequencing analyses, the selective pressure exerted by therapy allows resistant subclones to endure and expand while sensitive cells are eradicated, leading these resilient populations to dominate at relapse and drive a more aggressive, treatment-resistant disease phenotype [12]. Consequently, this pervasive heterogeneity profoundly constrains the durable effectiveness of therapeutic approaches targeting only one mechanism, including PARP inhibition or anti-angiogenic strategies.

OC presents a major diagnostic challenge, as its early symptoms, bloating, abdominal discomfort, and fatigue, are nonspecific and rarely prompt timely clinical investigation [13]. This problem is further intensified by the lack of effective screening modalities, with large randomized trials (PLCO, UKCTOCS) demonstrating that neither CA125 assessment nor transvaginal ultrasonography notably improves early-stage detection [14,15].

Standard treatment for advanced OC consists of optimal cytoreductive surgery followed by platinum and taxane chemotherapy. Although initial responses are common, recurrence occurs in the vast majority of patients with advanced HGSOC, typically within 2 or 3 years [16]. A major limitation of that approach is the development of platinum resistance, which emerges through diverse processes, ranging from augmented DNA-damage repair capacity and dysregulated drug transport to microenvironmental remodeling, metabolic reprogramming, or the survival of stem-like tumor subpopulations [17]. Analogous resistance pathways diminish the efficacy of taxanes, PARP inhibitors, and other targeted modalities, ultimately driving patients through successive treatment lines marked by waning therapeutic benefit and escalating toxicity [18,19].

The biological complexity of OC, coupled with its high mortality and the limitations of conventional therapies, accentuates a compelling imperative to pursue genuinely transformative therapeutic paradigms. Recent efforts have increasingly focused on immune-based strategies (including ICI, vaccines, cytokine therapy, CAR-T, and CAR-NK platforms), molecularly targeted agents (such as PARP, WEE1, ATR, and PI3K inhibitors), modulation of the tumor immune microenvironment, and rational therapeutic combinations that integrate chemotherapy or PARP inhibition with immunotherapy. Although the accelerating progress of immunological therapies holds meaningful promise for reshaping the clinical course of OC, therapeutic responses remain markedly variable, necessitating rigorous and integrative appraisal.

This review integrates clinical trial outcomes across immune checkpoint classes to identify reproducible patterns of efficacy, toxicity, and histologic specificity, providing a practical framework for future biomarker-driven trial design. Moreover, the review aims to highlight critical gaps in current knowledge that necessitate continued investigation. In light of the accelerating expansion of immunotherapeutic research and the growing body of clinical evidence, a rigorous, contemporary, and comprehensive synthesis is indispensable for clarifying both the transformative potential and the inherent constraints of emerging therapeutic avenues in OC.

## 2. Methods

A focused literature search was conducted across PubMed/MEDLINE, Embase, and ClinicalTrials.gov to identify relevant publications from the last six years. However, older studies were included selectively when considered essential for the biological context, landmark clinical evidence, or to support mechanistic explanations. Search terms included combinations of “ovarian cancer”, “epithelial ovarian cancer”, “immune checkpoint inhibitor”, “immunotherapy”, “PD-1”, “PD-L1”, “CTLA-4”, “TIM-3”, “nivolumab”, “pembrolizumab”, “avelumab”, “atezolizumab”, “durvalumab”, “ipilimumab”, “tremelimumab”, and “dostarlimab”. Additional records were identified through manual screening of reference lists from key review articles and major oncology conference proceedings.

Priority was given to prospective phase II and phase III clinical trials evaluating ICI as monotherapy or in combination with chemotherapy, targeted agents, anti-angiogenic therapy, or other immunotherapeutic strategies in EOC. Conference abstracts were included when they reported clinically relevant and recent data, particularly from ongoing or recently completed phase II/III trials, and were preferentially selected from major oncology meetings (e.g., ASCO, ESMO). Recently published phase III studies available during manuscript revision (e.g., KEYNOTE-B96) were incorporated to ensure up-to-date evidence.

Studies were included if they reported at least one clinically relevant efficacy endpoint (objective response rate, progression-free survival (PFS), overall survival (OS), or duration of response) and/or safety outcomes. Ongoing trials without available efficacy data were included only when they provided meaningful insight into emerging therapeutic strategies and were clearly identified as such in the text. Preclinical-only studies, case reports, non-English publications, and studies unrelated to ICI were excluded. Selection bias was minimized by prioritizing prospective clinical trials and cross-validating findings across multiple sources.

Given the heterogeneity of study designs, patient populations, and therapeutic regimens, formal meta-analysis was not performed. Instead, findings were synthesized narratively with emphasis on trial design, efficacy signals, safety profiles, and biological rationale. Publications were selected for their representativeness of current research directions and their relevance to clinical practice and the development of novel therapeutic strategies. This work represents a narrative (non-systematic) review.

Given the rapid evolution of immunotherapy in oncology, the present review is not exhaustive. Rather, its purpose is to provide a concise synthesis of the most important trends, findings, and limitations associated with the application of immunotherapy in OC. This paper aimed to discuss contemporary directions in immunotherapy development and their clinical significance, rather than to perform a quantitative assessment of the efficacy of specific interventions.

## 3. Clinical Evidence

### 3.1. PD-1 Immune Checkpoint Inhibition

#### Mechanism of PD-1 Immune Checkpoint Inhibition Therapy

Typically, the immune system possesses the capacity to recognize and eliminate cancer cells to a certain extent. The presence of tumor-infiltrating lymphocytes (TILs) has been associated with improved clinical outcomes across multiple malignancies, including OC [20]. Depending on TIL density within the tumor microenvironment, OCs are classified as immunologically “hot” tumors, characterized by prominent immune infiltration, or “cold” tumors, defined by relative immune exclusion [21]. Cytotoxic T lymphocytes, responsible for malignant cell destruction, require two distinct signals for activation. The first is an antigen-specific signal, mediated through the interaction between the T-cell receptor (TCR) and the major histocompatibility complex (MHC) peptide presented by antigen-presenting cells (APCs). The second is a co-stimulatory signal, conveyed through the engagement of co-receptors on T cells with their respective ligands on APCs. These can either promote T-cell activation, as in the case of stimulatory receptors such as CD28, or halt it, as observed with inhibitory receptors. Among the latter, PD-1 represents one of the most pivotal inhibitory co-receptors, playing a central role in cancer immunology [22].

**Figure 1:**
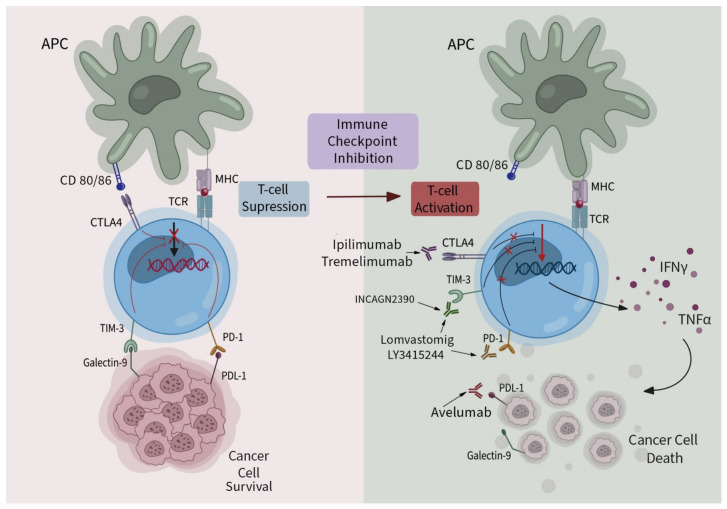
The mechanism of selected immune checkpoint inhibition strategies. Alt text: Schematic representation of the PD-1/PD-L1, CTLA-4/CD80/CD86, and TIM-3 signaling pathways and their roles in regulating T-cell activity within the tumor microenvironment. PD-1 binding to PD-L1 inhibits T-cell activation at the tumor site. CTLA-4 competes with CD28 for binding to CD80/CD86 on antigen-presenting cells (APCs), thereby suppressing early T-cell activation. TIM-3 interacts with ligands such as Galectin-9, contributing to T-cell exhaustion and immune suppression. Therapeutic antibodies targeting these pathways are shown at the corresponding interaction sites. Repeated elements (e.g., Galectin-9) indicate ligand–receptor interactions involved in inhibitory signaling. Abbreviations: APC, antigen-presenting cell; MHC, major histocompatibility complex; TCR, T-cell receptor; CTLA4, cytotoxic T-lymphocyte-associated protein 4; PD-1, programmed cell death protein 1; PD-L1, programmed death-ligand 1; TIM-3, T-cell immunoglobulin and mucin-domain containing-3; CD80/86, cluster of differentiation 80/86; IFNγ, interferon gamma; TNFα, tumor necrosis factor alpha.

PD-L1 (CD274, B7-H1) is physiologically expressed on B lymphocytes, dendritic cells (DCs), macrophages, and mast cells, similarly to PD-L2 (CD273, B7-DC), which is absent but inducible on DCs. Both PD-L1 and PD-L2 are type I transmembrane glycoproteins with IgV and IgC extracellular domains [23]. Their unregulated expression in solid malignancies, including OC, has been associated with elevated tumor aggressiveness and heightened mortality risk due to suppressed immune response. PD-1^+^ expression has been reported in approximately 93% of OC cases, whereas PD-L1^+^ expression is observed in about 40% [22]. Overexpression of both molecules together has been identified in over one-third of ovarian tumors, highlighting their contribution to disease progression [24]. Because the interaction between PD-1 and PD-L1 inhibits cytotoxic T cells, diminishes inflammation, and promotes tumor immune tolerance, therapeutic blockade of the PD-1/PD-L1 axis has the potential to restore T cell function, leading to the eradication of PD-1^+^/PD-L1^+^ cancerous cells [25]. The mechanism of all the mentioned ICI strategies is illustrated in Figure 1.

### 3.2. Latest Clinical Phase II Evidence for PD-1 Immune Checkpoint Inhibition

#### 3.2.1. PEACOCC (Pembrolizumab Monotherapy)

In the PEACOCC phase II trial, the target population consisted of women diagnosed with CCOC. Investigated therapy involved monotherapy with PD-1 inhibitor pembrolizumab, administered intravenously at a dose of 200 mg every three weeks for up to two years, with the option of a one-year retreatment period in the event of disease progression following the initial treatment course [26].

The results indicate that a group of patients achieved durable disease stabilization; although not all participants responded, those who did frequently experienced long-lasting, favorable effects. According to data published in *JAMA Oncology* in 2025, the 12-week PFS rate was 42%, with an overall response rate (ORR) of 25%, corresponding to 12 partial responses. Median PFS was found to be 2.7 months, while the median OS was 14.8 months. Additionally, 98% of tumors were proficient in mismatch repair (pMMR), which means that pembrolizumab remained clinically active despite the absence of microsatellite instability (MSI) or mismatch repair deficiency (dMMR) [26]. It shows that CCOC may respond to PD-1 inhibition independently of MSI/dMMR status, unlike many other tumor types, including HGSOC. This suggests that alternative mechanisms, such as high PD-L1 expression and specific genomic drivers in CCOC, may dictate sensitivity to PD-1 blockade.

The treatment was generally well tolerated. Grade 3 treatment-related adverse effects were present in 19% of patients, while grade 4 or 5 events remained unreported [26].

#### 3.2.2. NCT05296512 (Pembrolizumab + Lenvatinib)

In the phase II trial NCT05296512, involving recurrent CCOC patients, pembrolizumab therapy was combined with the anti-angiogenic tyrosine kinase inhibitor lenvatinib, which targets various signaling pathways, mainly VEGF. The treatment consisted of pembrolizumab administered intravenously every three weeks, together with daily oral lenvatinib with predefined dose reductions in the event of adverse reaction occurrence [27].

The obtained data revealed favorable anti-tumor activity, where 30% of patients achieved the desired response. The 6-month PFS rate was nearly 76%, with a median PFS of 10.9 months. Additionally, a substantial proportion of patients achieved disease stabilization.

The most common adverse effects included hypertension, fatigue, and hypothyroidism, often necessitating dose reductions in lenvatinib. The safety profile was consistent with that previously reported for levatinib therapies, with no unanticipated adverse events occurring [28]. Results indicate that the addition of lenvatinib may enhance antitumor activity and improve clinical outcomes in patients with CCOC. However, given the single-arm design of the study, these findings should be interpreted with caution. Direct comparisons with outcomes from other trials, including pembrolizumab monotherapy, are not methodologically appropriate due to differences in study populations and design. Therefore, the observed activity should be considered preliminary and requires confirmation in larger, randomized clinical trials.

#### 3.2.3. KEYNOTE-100 (Pembrolizumab Monotherapy)

KEYNOTE-100 is one of the largest phase II trials evaluating the effectiveness of pembrolizumab monotherapy in recurrent, advanced OC, encompassing two independent cohorts of patients previously treated with multiple lines of systemic therapy. Participants received pembrolizumab at a dose of 200 mg intravenously every three weeks until disease progression or the emergence of unacceptable toxicity. The primary objective of the study was to determine the ORR and to assess the relationship between PD-L1 expression levels and treatment efficacy.

The findings demonstrated modest clinical activity, with an ORR of 8.0% in the first cohort and 9.9% in the second. A clear association between PD-L1 expression, measured by the Combined Positive Score (CPS), and therapeutic efficacy was observed, with the greatest activity occurring in patients with CPS ≥10. The median PFS was approximately 2.1 months, while the OS ranged from 17.6 to 18.7 months. These results underscore the limited yet clinically meaningful activity of pembrolizumab in a heavily pretreated patient population [29].

The safety profile was consistent with previously reported experience for pembrolizumab. Adverse events were predominantly characterized by fatigue, nausea, and immune-mediated toxicities, and grade ≥3 events occurred in only a small proportion of patients. This study provides an important foundation for the ongoing development of immunotherapy in OC, offering evidence of anti-PD-1 activity in a selected subset of patients and underscoring the relevance of biomarkers such as CPS in guiding therapeutic decision-making [29].

#### 3.2.4. MoST-CIRCUIT (Nivolumab + Ipilimumab)

In the multicenter, prospective Phase II MoST-CIRCUIT trial (NCT04969887), the clinical activity of dual ICI with nivolumab (anti-PD-1) and ipilimumab (anti-CTLA-4) was evaluated in patients with advanced CCOC and clear-cell endometrial carcinoma (CCEC). Eligible patients had received no more than one prior line of systemic therapy. Treatment consisted of nivolumab combined with ipilimumab administered every three weeks for four induction cycles, followed by maintenance nivolumab every four weeks until disease progression or unacceptable toxicity.

The combination demonstrated substantial antitumor activity. The ORR was 54%, including 12% complete responses (CR) and 42% partial responses (PR). In the CCOC cohort, the ORR reached 55%, indicating pronounced sensitivity of this histologic subtype to combined PD-1 and CTLA-4 inhibition. Responses were durable, with the median duration of response not reached at the time of data cutoff. The 6-month PFS rate was 58%, while median PFS and OS had not yet been reached, reflecting ongoing clinical benefit in a substantial proportion of patients.

Treatment-related adverse events were consistent with the known toxicity profile of dual checkpoint inhibition. Grade 3–4 immune-related adverse events occurred in approximately 35% of patients, most commonly involving gastrointestinal, endocrine, and hepatic systems. One treatment-related fatal case of immune-mediated myocarditis was reported, underscoring the need for careful patient selection and vigilant toxicity monitoring [30].

Collectively, these findings indicate that dual PD-1/CTLA-4 blockade yields markedly higher response rates in CCOC than those historically observed with PD-1 monotherapy, supporting the concept that intensified ICI may partially overcome the immunologically “cold” microenvironment characteristic of this subtype. Although limited by its single-arm design, the MoST-CIRCUIT trial provides compelling evidence to justify further investigation of this combination in randomized Phase III studies and positions CCOC as a particularly promising target for immune-based combination strategies. All mentioned phase II clinical trials of PD-1-based ICI in OC are summarized in Table 1.

Taken together, phase II studies of PD-1–based therapies in ovarian cancer demonstrate modest and heterogeneous clinical activity, with response rates generally limited in unselected populations. Notably, more favorable outcomes are observed in biologically distinct subgroups, particularly clear cell ovarian carcinoma, suggesting that tumor histology and underlying molecular features significantly influence responsiveness to immune checkpoint inhibition. These findings highlight the importance of patient selection and provide a rationale for further biomarker-driven investigation.

### 3.3. Latest Clinical Phase III Evidence for PD-1 Immune Checkpoint Inhibition

The ATHENA-COMBO trial investigated the potential benefit of adding immunotherapy to rucaparib, a PARP inhibitor, in the disease maintenance conditions. The patient group consisted of women with newly diagnosed OC who achieved either a complete or partial response from first-line platinum-based chemotherapy. Patients were randomized to receive either rucaparib, as monotherapy, or a combination of rucaparib with the PD-1 inhibitor nivolumab. The treatment regimen in the experimental setting included oral administration of rucaparib twice daily, coupled with nivolumab intravenously every four weeks, while the other group of patients received rucaparib alone [31].

Unexpectedly, clinical outcomes revealed that the addition of nivolumab not only did not improve PFS but was in fact associated with inferior PFS values, compared to rucaparib monotherapy. Experimental therapy achieved a median PFS equal to 15 months, compared to 20.2 months with rucaparib alone. Furthermore, there was no observed benefit in OS in patients who received the rucaparib and nivolumab combination [31].

Although the incidence of treatment-related adverse events of grade three was similar across groups, specific conditions were more frequently observed with the combination. Most notably, neutropenia occurred in 25.4% of patients in the combination group vs. 15.4% with monotherapy, and elevations in ALT/AST in 21.2% vs. 10.0%, respectively. Additionally, patients in the nivolumab arm experienced shorter median treatment exposure, consistent with the inferior PFS observed [31].

This trial provided no evidence of clinical value from combining immunotherapy with rucaparib in the first-line maintenance setting. Furthermore, the results advocate that this combination may have an unfavorable effect, as it was related to reduced PFS compared with the PARP inhibitor alone.

#### 3.3.1. FIRST (Dostarlimab + Chemotherapy → Niraparib Maintenance)

The FIRST/ENGOT-OV44 trial investigated the effect of adding the PD-1 inhibitor dostarlimab to typical first-line therapy in patients with newly diagnosed, advanced OC. The participant group consisted of women who had undergone a cytoreductive surgery followed by platinum-based chemotherapy. Patients were randomized to receive chemotherapy plus dostarlimab followed by maintenance with niraparib and dostarlimab (with or without bevacizumab, at the discretion of the treating center) or to the control arm of chemotherapy alone followed by maintenance niraparib (with or without bevacizumab).

The treatment protocol included dostarlimab intravenously every 3 weeks in combination with chemotherapy for four cycles, followed by a maintenance dose every 6 weeks. Niraparib was administered with an individualized starting dose. Bevacizumab could be added to either group, according to institutional standards [32].

Results have shown a limited improvement in PFS. Median PFS was 20.6 months in the dostarlimab group versus 19.2 months in the control condition. However, this slight gain in PFS was not accompanied by a statistically significant difference in OS at 57% data maturity.

With respect to safety, the combination was typically well tolerated, with any adverse events being consistent with the previously established toxicity profiles of the individual agents, without new safety signals reported [32].

This specific study demonstrated that the incorporation of dostarlimab to standard chemotherapy resulted in only a slight PFS improvement of limited clinical significance. Accordingly, these data imply that combining PD-1 inhibition into the first-line setting does not substantially improve outcomes in patients with advanced OC.

#### 3.3.2. KEYNOTE-B96 (Pembrolizumab + Paclitaxel ± Bevacizumab)

The KEYNOTE-B96/ENGOT-ov65 trial was designed to further review the efficacy of PD-1 blockade in the context of platinum-resistant recurrent OC. Women qualified for enrolment were randomized to receive either chemotherapy with weekly paclitaxel, with or without bevacizumab, in combination with pembrolizumab, or the same dosing strategy with placebo instead of pembrolizumab. The study demonstrated a significant improvement in PFS in the combination arm, with benefit observed both in the overall population and in the PD-L1-positive subgroup [33]. A clinically meaningful OS benefit was also reported in the PD-L1-positive subgroup. Importantly, these results derive from a recently published phase III trial, providing robust evidence of the activity of immune checkpoint inhibition in a biomarker-defined population [34].

However, OS outcomes in the overall study population remain less clearly defined and require further follow-up for definitive interpretation. These findings suggest that pembrolizumab, in combination with paclitaxel ± bevacizumab, may represent a promising therapeutic option in platinum-resistant ovarian cancer, particularly in selected patients, and underscore the importance of biomarker-driven treatment strategies.

#### 3.3.3. JAVELIN Ovarian 100 (Carboplatin/Paclitaxel ± Avelumab)

In the global JAVELIN Ovarian 100 trial (NCT02718417), the therapeutic value of PD-L1 blockade with avelumab was investigated in previously untreated stage III–IV EOC, fallopian tube, or primary peritoneal cancer. The study tested whether introducing ICI either concurrently with platinum–taxane chemotherapy and continued as maintenance, or as maintenance alone after chemotherapy, could prolong PFS relative to standard chemotherapy followed by observation. Patients were randomized (1:1:1) to: (A) carboplatin (AUC 5–6) plus paclitaxel for six cycles followed by observation, (B) the same chemotherapy followed by avelumab maintenance, or (C) chemotherapy plus avelumab, followed by avelumab maintenance [35].

At the prespecified interim analysis (data cutoff 7 September 2018), futility boundaries for PFS were crossed, and the trial was discontinued. Median PFS was 16.8 months in the chemotherapy + avelumab maintenance arm, 18.1 months in the chemotherapy + avelumab + avelumab arm, and not estimable in the control arm. As efficacy follow-up was halted at futility, OS was not further assessed as a definitive endpoint in this report [35].

The safety profile revealed no unexpected signals beyond those anticipated for chemotherapy and ICI. The most frequent grade 3–4 adverse events included neutropenia, anemia, and decreased neutrophil counts, while serious adverse events were reported more often in the avelumab-containing arms than in the control group. Overall, the trial provided decisive evidence that frontline incorporation of PD-L1 inhibition with avelumab, either as maintenance or in combination with chemotherapy, does not improve outcomes in unselected patients with newly diagnosed advanced EOC, reinforcing the need for strategies based on biomarker specificity and alternative combinatorial backbones.

#### 3.3.4. JAVELIN Ovarian 200 (Avelumab vs. PLD vs. Avelumab + PLD)

JAVELIN Ovarian 200 was the first randomized phase III trial to compare PD-L1 inhibition, either as avelumab monotherapy or in combination with pegylated liposomal doxorubicin (PLD), with standard PLD chemotherapy in patients with platinum-resistant OC. Participants received avelumab every two weeks, either alone or combined with PLD every four weeks, while the control arm was treated with standard PLD.

The study results did not demonstrate an improvement in either PFS or OS in the monotherapy or combination arms. Median PFS was 1.9 months with avelumab, 3.7 months with avelumab plus PLD, and 3.5 months with PLD alone. Median OS similarly failed to show benefit, reaching 11.8 months in the avelumab arm, 15.7 months in the combination arm, and 13.1 months in the PLD group. OORs were low across all treatment arms and did not exceed those observed with PLD alone.

The safety profile was consistent with prior experience, with most adverse events being grade 1–2 in severity; the most common toxicities included nausea, fatigue, and dermatologic reactions. Collectively, the findings of JAVELIN Ovarian 200 demonstrated that neither PD-L1 inhibitor monotherapy nor its combination with chemotherapy provides clinical benefit in platinum-resistant OC, thereby shaping the subsequent direction of research involving avelumab [36].

#### 3.3.5. IMagyn050 (Atezolizumab + Bevacizumab + Chemotherapy)

IMagyn050 was the largest phase III immunotherapy trial conducted to date in OC, enrolling more than 1300 patients with newly diagnosed, advanced disease. The therapeutic backbone consisted of standard carboplatin paclitaxel chemotherapy combined with bevacizumab, while the experimental arm incorporated atezolizumab administered every three weeks in addition to this regimen. The study also included comprehensive biomarker analyses, including assessments of PD-L1 expression and BRCA/HRD status.

The trial results demonstrated no improvement in PFS in the overall study population or within biomarker-defined subgroups. Median PFS was 19.5 months in the atezolizumab arm compared with 18.4 months in the control arm, a difference that did not reach statistical significance. OS likewise did not improve in subsequent interim analyses. ORRs were comparable between the treatment arms, and no clinical benefit was observed even among patients with high PD-L1 expression.

The regimen was generally well tolerated, with a safety profile consistent with the known toxicities of atezolizumab and bevacizumab. IMagyn050 represents one of the most important negative studies in the field of OC immunotherapy and provided decisive evidence that the addition of PD-L1 blockade to first-line standard therapy does not confer clinical benefit [37]. All mentioned phase III clinical trials of PD-1/PD-L1 ICI strategies in OC are summarized in Table 2.

In contrast to early-phase studies, the majority of phase III trials have failed to demonstrate a consistent survival benefit with the addition of immune checkpoint inhibitors in unselected ovarian cancer populations. These results suggest that empiric incorporation of ICIs into standard treatment regimens is insufficient and underscore the need for more precise, biomarker-driven approaches and biologically informed combination strategies.

### 3.4. CTLA-4 Pathway Inhibition

#### Mechanism of CTLA-4 Pathway Inhibition Therapy

CTLA-4 is a critical immune checkpoint that guarantees tolerance and prevents autoimmunity. Its expression is not limited to T cells; on B-1a cells, CTLA-4 is critical for sustained immune tolerance, and its absence leads to autoantibody production, therefore highly influencing autoimmune pathology [38]. At the molecular level, the cytoplasmic domain of CTLA-4 orchestrates the induction of regulatory T cells (Tregs) and attenuates autoimmunity through the upregulation of Foxp3 expression [39]. Engaging B7 ligands with greater acidity than CD28, CTLA-4 delivers inhibitory signals that counteract activation driven by CD28, restricting T cell proliferation and preventing excessive immune response.

In OC, particularly HGOC, CTLA-4 functions dually as a guardian of immune homeostasis and a mediator of immunosuppression associated with tumors. Although elevated CTLA-4 expression fosters an immunosuppressive environment, its co-expression with other inhibitory receptors, such as PD-1 and VISTA, associates with a more permissive immune context and enhanced survival, suggesting these tumors have the potential of being distinctly amenable to checkpoint blockage [40]. Mechanistic studies reveal that cyclooxygenase-2 (COX-2) overexpression potentiates CTLA-4 action in the context of HGOC. Notably, synchronous COX-2 inhibition with CTLA-4 blockage restores NK cell cytotoxicity, promoting tumor elimination [41]. Genetic variation, in addition to expression patterns, further underscores CTLA-4 signaling in the pathogenesis of OC. The scientific data indicate that certain CTLA-4 polymorphisms confer elevated susceptibility to EOC [42]. Furthermore, transcriptomic analyses imply that high CTLA-4 expression is in accordance with ameliorated outcomes in patients treated with ICI. Collectively, these findings position CTLA-4 signaling as a principal determinant of both ovarian tumor evolution and therapeutic outcomes [43].

### 3.5. Latest Clinical Phase II Evidence for CTLA-4 Pathway Inhibition

#### 3.5.1. NRG-GY003 (Nivolumab ± Ipilimumab)

Randomized NRG-GY003 study investigated the therapeutic value of combining nivolumab with Ipilimumab in patients diagnosed with either recurrent or persistent EOC, fallopian tube, or primary peritoneal carcinoma. The cohort comprised patients with a platinum-free interval of less than twelve months, displayed a measurable disease as defined by RECIST 1.1, and had undergone one to three prior regimens. The trial juxtaposed PD-1 inhibition alone with integrated PD-1 and CTL-4 suppression, with the primary endpoint being OR within six months of therapy.

At that time, the combination of nivolumab and ipilimumab reached a response rate of 31.4% compared with 12.2% isolated nivolumab therapy (odds ratio 3.28; *p* = 0.034). Likewise, PFS was superior in the experimental arm, with a median of 3.9 months versus 2.0 months in the nivolumab group. No statistically significant OS benefit was observed.

Patients in the isolated PD-1 inhibition received nivolumab 3 every two weeks, while those in the integrated therapeutic approach received nivolumab plus ipilimumab every three weeks for four induction cycles, followed by nivolumab every two weeks as maintenance. Dual ICI was associated with a higher incidence of treatment-related toxicity, with grade greater than three adverse events affecting 49% of patients versus 33% in the nivolumab group. It is worth emphasizing that no deaths related to treatment were noted [44].

Collectively, the addition of CTLA-4 inhibition did in fact improve the response rate and relatively prolonged PFS; however, at the cost of amplified harmfulness, so its incorporation should be further studied in phase III trials to evaluate the clinical efficacy of the therapy.

#### 3.5.2. BrUOG 354 (Nivolumab vs. Nivolumab + Ipilimumab in Clear-Cell Gynecologic Carcinomas)

Another randomized study on the clinical benefit of nivolumab combination with ipilimumab focused on patients with previously treated clear-cell gynecologic carcinomas, including CCOC and CCEC. The endpoints of the BrUOG 354 study included PFS, OS, and the ORR.

According to the clinical outcome, presented at ASCO 2024, the combination cohort demonstrated a median PFS of 5.6 months compared with 2.2 months for nivolumab alone, and a median OS of 24.7 months versus 17.3 months, respectively. As anticipated, experimental group therapy has caused a higher incidence of grade greater than three treatment-related adverse events compared to patients treated with monotherapy, perpetuating previously established safety concerns distinctive for dual PD-1/CTLA-4 inhibition [45].

Participants in both study groups were treated with nivolumab intravenously every two weeks, while those in the combination arm also underwent treatment with ipilimumab every six weeks [45]. In aggregate, these results indicate that nivolumab plus ipilimumab therapy offers clinically meaningful improvements in PFS and shows a signal for benefit in OS compared with nivolumab alone in patients with clear-cell histology, albeit at the cost of increased toxicity.

#### 3.5.3. NCT03026062 (Tremelimumab ± Durvalumab)

NCT03026062 randomized trial aimed to examine the clinical outcome of tremelimumab given either sequentially with durvalumab or in combination, in women with heavily pretreated, HGSOC resistant to platinum therapy. The primary endpoint for this study was PFS examination with the intention of determining whether dual ICI could improve outcomes compared with a sequential strategy [46].

The outcomes displayed no striking difference in median PFS between the combination and sequential cohorts. Likewise, no clear OS advantage was noted, reflecting the limited clinical activity of this approach in this patient population. No unusual safety concerns were observed.

Therapeutic regimen for patients in the sequential therapeutic strategy received tremelimumab every four weeks for four doses, followed by durvalumab every four weeks. In the combination setting, tremelimumab was administered together with durvalumab every four weeks, followed by durvalumab maintenance every four weeks [46].

The results collectively indicate that the addition of PD-L1-inhibiting factor to CTLA-4 therapy did not confer any clinical benefits in this refractory setting. Collected data do not support the routine use of this combination in the case of HGSOC.

#### 3.5.4. KGOG-3046 TRU-D (Neoadjuvant Chemotherapy + Durvalumab + Tremelimumab)

Incorporation of dual ICI with durvalumab (PD-L1) and tremelimumab (CTLA-4) to standard neoadjuvant chemotherapy in newly diagnosed women with stage IIIC-IVB EOC. Patients meeting eligibility criteria received preoperative paclitaxel and carboplatin, subsequent to which they underwent cytoreductive surgery, to evaluate the potential of integrating ICI into first-line treatment, with the aim of elevating the 12-month PFS value.

The encouraging outcome showed a 12-month PFS reaching 63.6%, with 24-month and 30-month rates of 45.0% and approximately 40%, respectively. Interval-debuting surgery yielded a remarkably high rate of R0 resections, highlighting a compelling synergism between immunotherapeutic and chemotherapeutic modalities, thereby suggesting strategic augmentation of surgical performance. Importantly, this was a small, non-randomized phase II study without a comparator arm, which limits the strength of efficacy interpretation.

Nonetheless, the trial demonstrated the feasibility of incorporating dual immunotherapy into induction chemotherapy without prohibitive toxicity [47]. In the original study population, patients were given durvalumab every three weeks, combined with tremelimumab alongside each of the three neoadjuvant chemotherapy cycles. In the expansion cohort, the regimen was adjusted to a single priming dose of tremelimumab combined with durvalumab, followed by standard preoperative chemotherapy per protocol [47].

TRU-D revealed that incorporating durvalumab and tremelimumab to neoadjuvant chemotherapy was viable, produced promising PFS rates, and improved surgical outcomes. While optimistic, these findings remain exploratory and require confirmation in a randomized setting before clinical adoption.

#### 3.5.5. NCT02571725 (Tremelimumab ± Olaparib)

Last but not least, a trial exploring the potential of CTLA-4 blockade with tremelimumab, either alone or in combination with the PARP inhibitor, olaparib, was performed on patients with recurrent OC that successfully responded to platinum treatment. This time, the study investigated whether combining immunotherapy with DNA damage repair inhibition could enhance antitumor activity in the case of OC, proposing a novel strategy of CTLA-4 combination.

Early reports evidence a clinical benefit rate of nearly 46%, advocating for impactful activity [48]. However, specific data on PFS and OS have not yet been fully reported, leaving the effectiveness of this strategy incomplete at this stage.

Tremelimumab was administered in accordance with established schedules, with olaparib given in combination in one study arm. The exact regimen varied by cohort and has not been comprehensively described in preliminary reports. Adverse events consistent with tremelimumab were observed, and the combination with olaparib appeared tolerable without introducing unusual symptoms [49].

Available data attest to the operational viability of simultaneous CTLA-4 and PARP signaling disruption, providing an incipient yet persuasive signal of therapeutic engagement. A full appraisal of the therapeutic possibilities will, however, depend on the publication of mature survival trends and comprehensive efficacy analyses. All mentioned phase II clinical trials of CTLA-4-based checkpoint inhibition strategies in OC are summarized in Table 3.

Overall, dual checkpoint blockade targeting PD-1 and CTLA-4 appears to enhance response rates compared with monotherapy, particularly in selected subgroups such as clear-cell histology. However, these benefits are accompanied by substantially increased toxicity and have not consistently translated into improved survival outcomes, highlighting the need to better define the patient populations most likely to benefit from this approach.

### 3.6. TIM-3 Signaling Blockade

#### Mechanism of TIM-3 Signaling Blockade Therapy

TIM-3, first identified on Th1 CD4^+^ T cells and CD8^+^ cytotoxic T lymphocytes producing interferon-γ. Additionally, it has also been identified on NK cells, Th17 cells, γδ T cells, regulatory T cells, macrophages, DCs, and mast cells, underscoring its wide immunoregulatory role [50]. In human organisms, TIM-3 is a part of the TIM family, which, apart from TIM-3, also includes TIM-1 and TIM-4, and is encoded by HAVCR2 [51]. Several ligands have been identified for TIM-3, each producing characteristic signals that regulate immune responses. Galectin-9 induces apoptosis in Th1 and CD8^+^ T cells, thereby inducing T-cell exhaustion [52]. Phosphatidylserine, translocated to the outer membrane during apoptosis, interacts with TIM-3 to facilitate antigen presentation to cytotoxic T cells by DCs, with concurrent inhibition of T-cell and NK-cell activity [53]. HMGB-1 engagement prevents DCs from sensing nucleic acids, which diminishes immune recognition of genetic material associated with tumors [54]. Furthermore, TIM-3 binding to CEACAM-1 expressed on T cells intensifies the negative modulation of their function [55].

Consequently, inhibition of TIM-3 prevents suppression mediated by ligands, sustaining TCR and costimulatory signaling. Such a condition revitalizes immune function, restoring activity in exhausted CD8^+^ T cells and NK cells [56]. The presence of TIM-3 together with PD-1 on exhausted T cells supports therapeutic strategies that concurrently address both checkpoints to counteract resistance to PD-1/PD-L1 blockade.

### 3.7. Latest Clinical Phase II Evidence for TIM-3 Signaling Blockade

#### 3.7.1. Lomvastomig (PD-1×TIM-3 Bispecific)

Lomvastomig constitutes the translational debut of a specific antibody simultaneously engaging PD-1 and TIM-3. An ongoing Phase I investigation, employing stepwise dose increments and expansion arms, has accrued individuals with advanced or metastatic solid malignancies, including OC. Results to date remain preliminary, with no ovarian-specific outcomes yet reported. Likewise, efficacy rates are immature, and PFS or OS projections have not been presented. Additionally, recommended dose levels for expansion are being established. Up till now, no unusual adverse events that were inconsistent with those expected with checkpoint inhibition have been observed [57]. Taken together, lomvastomig embodies a mechanistically grounded bispecific checkpoint strategy under initial clinical evaluation, though its definite therapeutic impact in OC remains to be validated.

#### 3.7.2. INCAGN02390 (Anti-TIM-3 Antibody)

Clinical evaluation of INCAGN02390, a selective TIM-3 antibody, has been conducted in patients with advanced or metastatic solid tumors who had received multiple prior therapies (60% had received ≥3 prior lines; 48% had prior ICI exposure), with OC encompassed within the eligible tumor spectrum. Intravenous doses every two weeks were assessed in an escalation study. Among participants, one PR was observed (2.5%), yielding a disease control rate of 18%, reflecting a modicum of activity with monotherapy. Median PFS was 1.9 months, while OS data remain immature. The maximum tolerated dose was not reached, while adverse events linked to therapy were reported in 30% of patients, with less than 8% being grade three or higher. A recommended Phase II dose of 400 mg IV every two weeks was established, intended for future combination strategies [58]. Collectively, the findings demonstrated an acceptable tolerability yet limited antitumor activity infused as a sole agent, reinforcing the rationale for multimodal approaches in OC and other solid tumors.

#### 3.7.3. LY3415244 (TIM-3×PD-L1 Bispecific)

LY3415244, an antibody engineered for dual ICI of TIM-3 and PD-L1, has undergone initial clinical investigation in advanced solid tumors, including OC. Enrolled participants were administered varying doses of LY3415244 intravenously, once every two weeks. The primary objectives of the study were safety and immunogenicity. Efficacy was limited to a single unconfirmed PR in a patient with non–small-cell lung cancer, previously treated with PD-1 inhibitors. The trial was terminated early owing to excessive immunogenicity, with anti-drug antibodies detected in 100% of the studied population and anaphylactic infusion reactions occurring in two. Consequently, PFS and OS were impossible to establish [59]. The trial illustrates both the therapeutic potential and the liabilities of bispecific strategies; in this instance, immunogenicity rendered the molecule clinically untenable. All mentioned phase II clinical trials targeting TIM-3 in OC are summarized in Table 4.

Although early-phase studies suggest that TIM-3 targeting is biologically relevant and generally well tolerated, its clinical activity as a single agent remains uncertain. Importantly, most ongoing trials are still in early phases, and robust efficacy data are not yet available, limiting definitive conclusions regarding its therapeutic potential. At present, TIM-3 should be considered an exploratory target, with its future role likely dependent on combination strategies and more precise patient selection. Further clinical data will be required to determine whether TIM-3 inhibition can translate into meaningful clinical benefit.

## 4. Discussion

### 4.1. Cross-Strategy Comparison of Immune Checkpoint Inhibition Approaches in Ovarian Cancer

Across immune checkpoint therapeutic strategies evaluated in OC, outcomes align along an efficacy-toxicity continuum, evident when PFS is assessed in parallel with the incidence of grade ≥3 adverse events. Notably, observed outcomes are highly contingent upon histologic subtype and disease context, underscoring the heterogeneity of therapeutic benefit and risk across OC populations. PD-1/PD-L1 monotherapy, as exemplified by trials such as PEACOCC in CCOC and KEYNOTE-100 in recurrent HGSOC, demonstrated the most favorable safety profile among clinically mature immunotherapeutic approaches, with severe toxicities generally reported in fewer than one quarter of treated patients. Nevertheless, this tolerability was accompanied by limited efficacy, as median PFS typically ranged between approximately two and three months across heavily pretreated populations, particularly in HGSOC. Attempts to augment antitumor activity through immune intensification using dual ICI incorporating CTLA-4, especially combinations of nivolumab and ipilimumab evaluated in NRG-GY003, MoST-CIRCUIT, and BrUOG 354, have demonstrated reproducible improvements in clinical outcomes. These benefits were particularly evident in biologically distinct subgroups, including CCOC and other clear-cell gynecologic malignancies, in which median PFS extended into the four to six-month range and response rates substantially exceeded those observed with PD-1 monotherapy. However, these efficacy gains were consistently accompanied by a pronounced increase in immunogenic toxicity, with grade ≥3 adverse events reported in approximately one third to one half of treated patients, thereby limiting the clinical applicability of this approach to carefully selected populations and predominantly to controlled clinical trial settings. By contrast, strategies pairing PD-1 blockade with anti-angiogenic therapy, as in NCT05296512, where pembrolizumab plus lenvatinib combination was utilized in recurrent or persistent CCOC, produced the most substantial prolongation of PFS observed in this analysis, with median PFS approaching 11 months. These findings point to a potentially synergistic interplay between vascular remodeling and immune activation in this particular subtype. These observations must be interpreted in light of the absence of consistently reported rates of high-grade toxicity in the available data, which precludes robust quantitative comparison with other ICI attempts and confines safety assessment largely to qualitative interpretation. By contrast, combinations of PD-1 blockade with PARP inhibition or cytotoxic chemotherapy, including ATHENA-COMBO and FIRST in the first-line or maintenance setting for predominantly HGSOC, as well as IMagyn050 and JAVELIN Ovarian 100 in newly diagnosed advanced EOC, failed to deliver consistent or clinically meaningful improvements in PFS. Additionally, these strategies were frequently associated with increased hematologic and systemic toxicity, reinforcing an unfavorable balance between hazards and potential benefits in largely unselected patient populations. At the opposite end of the therapeutic landscape, therapy approaches directed against TIM-3, including INCAGN02390 monotherapy and early-phase bispecific agents such as lomvastomig and LY3415244 in heavily pretreated, chiefly SOC, low rates of grade ≥3 adverse events, often below 10%, but minimal clinical activity, with median PFS of approximately two months and no clear evidence of durable disease control. The lack of efficacy observed in multiple phase III trials, including IMagyn050 and JAVELIN Ovarian 100/200, can be interpreted in the context of both biological and design-related limitations. In particular, the absence of biomarker-driven patient selection likely diluted potential treatment effects in unselected populations, while the predominantly immunosuppressive and immune-excluded tumor microenvironment characteristic of HGSOC further limits responsiveness to ICI. In addition, the use of empiric combination strategies without a clear mechanistic rationale may have contributed to the inconsistent outcomes observed across trials. These findings collectively highlight that both underlying tumor biology and suboptimal trial design are key contributors to the limited clinical benefit of ICIs in ovarian cancer. Taken together, these data delineate a central tension in OC immunotherapy, demonstrating that intensification of ICI can yield incremental gains in disease control within biologically defined subsets, most notably in CCOC, yet these benefits are inextricably linked to heightened toxicity, while more tolerable strategies remain constrained by limited efficacy. Resolving this dichotomy will require approaches guided by histologic context and biomarker stratification, together with mechanistically rational combination strategies, to meaningfully optimize the therapeutic index of immunotherapy in OC. Cross-strategy comparison of ICI approaches in OC is summarized in Table 5.

### 4.2. Biomarkers of Response and Resistance to Immune Checkpoint Inhibition in Ovarian Cancer

The inconsistent clinical efficacy of ICIs in OC has underscored the need for biomarkers that can inform patient selection and therapeutic strategy. Unlike malignancies in which single genomic alterations reliably identify candidates for immunotherapy, OC lacks a dominant predictive biomarker, reflecting its molecular heterogeneity and frequently immunosuppressive tumor microenvironment. Current evidence supports a clinically oriented, multifactorial model in which histologic subtype, immune infiltration, and selected molecular features collectively influence the likelihood of benefit from ICI.

#### 4.2.1. PD-L1 Expression and Combined Positive Score

PD-L1 expression is the most routinely assessed biomarker in clinical practice for PD-1/PD-L1 blockade. PD-L1 positivity is observed in approximately 40% of EOC [60]. Across trials, PD-L1-positive tumors show somewhat higher response rates than PD-L1-negative disease; however, most PD-L1-positive patients do not respond, and responses can occur in PD-L1-negative tumors [61]. Variability in scoring systems (TPS versus CPS), lack of assay harmonization, and intratumoral heterogeneity limit the clinical reliability of PD-L1 as a biomarker in patient selection for immunotherapy in OC [61,62].

#### 4.2.2. Microsatellite Instability and Mismatch Repair Deficiency

MSI-H/dMMR status is a validated, tumor-agnostic biomarker for immunotherapy responsiveness. In OC, MSI-H/dMMR is detected in only 3.9% of cases, primarily within serous, endometrioid, and clear cell carcinomas [63]. These tumors exhibit increased immune infiltration and frequently respond to ICI, supporting routine testing to identify rare candidates [64]. Nevertheless, MSI-H/dMMR accounts for only a small fraction of OCs and does not address the broader population that demonstrates primary resistance.

#### 4.2.3. Homologous Recombination Deficiency and BRCA Mutations

HRD and BRCA mutations are present in about 50% of HGSOC and are established predictors of response to PARP inhibitors, but do not reliably predict benefit from ICI monotherapy [65]. Although HRD-positive tumors may display increased immune signaling, clinical trials have not demonstrated consistent immunotherapy sensitivity based on HRD status alone [66]. From a clinical standpoint, HRD status may be more relevant for identifying patients who could benefit from PARP inhibitor-based combination strategies rather than guiding ICI use.

#### 4.2.4. Clear-Cell Histology

Histologic subtype represents one of the most clinically meaningful predictors of immunotherapy activity in OC. CCOC is associated with higher PD-L1 expression, increased TIL density, and reproducibly higher response rates to ICIs and dual checkpoint blockade compared with HGSC [67]. These findings support preferential enrollment of patients with clear-cell histology into immunotherapy trials and consideration of immunotherapy-based strategies in this subgroup.

#### 4.2.5. Tumor-Infiltrating Lymphocytes

High CD8^+^ TIL density correlates with improved prognosis and increased likelihood of response to ICIs. The presence of exhausted CD8^+^PD-1^+^ T cells suggests a pre-existing antitumor immune response that may be reactivated by checkpoint blockade [68]. Conversely, tumors lacking meaningful immune infiltration rarely respond. Assessment of immune infiltration may therefore assist in stratifying patients for immunotherapy-based approaches.

#### 4.2.6. Angiogenic and Immunosuppressive Signatures

VEGF-driven angiogenesis contributes to immune exclusion and resistance to ICIs [69]. Clinically, this provides a rationale for combining anti-angiogenic agents with ICIs to enhance immune cell trafficking and therapeutic efficacy. Early-phase clinical data supporting such combinations highlight the relevance of angiogenic signatures when selecting patients for combination strategies. Biomarkers of response and resistance to ICI in OC are represented in Table 6.

#### 4.2.7. Integrated Clinical Perspective

Collectively, available evidence indicates that no single biomarker reliably predicts response to ICI in OC. PD-L1 expression, MSI-H/dMMR status, and HRD provide limited standalone guidance for patient selection, while tumor mutational burden has minimal clinical utility in this disease. In contrast, histologic subtype, particularly clear cell ovarian carcinoma, and the presence of a pre-existing immune-inflamed microenvironment characterized by high CD8^+^ TIL density emerge as the most informative indicators of potential benefit. Angiogenic signatures further contribute to resistance and support the use of anti-angiogenic-immunotherapy combinations in selected settings. These observations underscore the need for integrated, multiparametric biomarker strategies and histology-informed trial designs to optimize patient selection and improve the therapeutic index of ICI in OC.

#### 4.2.8. Why Ovarian Cancer Is Resistant to Immune Checkpoint Inhibition

Ovarian cancer (OC), particularly high-grade serous ovarian cancer (HGSOC), is characterized by a strongly immunosuppressive tumor microenvironment (TME) that limits the efficacy of immune checkpoint inhibitors (ICIs). The TME is enriched with regulatory T cells (Tregs; FOXP3+), M2-polarized tumor-associated macrophages (TAMs), and myeloid-derived suppressor cells (MDSCs), which suppress cytotoxic T lymphocyte (CTL) activity and promote immune tolerance [70]. Immunosuppressive cytokines such as IL-10, TGF-β, and VEGF further induce T-cell exhaustion and inhibit dendritic cell (DC) maturation and antigen presentation. VEGF additionally contributes to abnormal angiogenesis and dysfunctional vasculature, thereby restricting immune-cell infiltration into tumors [70,71].

A further contributor to ICI resistance is the generally low-to-moderate tumor mutational burden (TMB) of OC, which limits neoantigen generation compared with more immunogenic tumors such as melanoma and lung cancer [72]. Although homologous recombination deficiency (HRD), especially in BRCA1/2-mutated tumors, is associated with increased neoantigen burden and tumor-infiltrating lymphocytes (TILs), most ovarian cancers remain homologous recombination proficient and immunologically “cold” [73]. Tumor-intrinsic mechanisms also contribute to immune escape, including loss of heterozygosity (LOH) at the HLA locus, which impairs antigen presentation, and foldback inversion (FBI)-associated tumors characterized by TGF-β signaling and immune exclusion phenotypes with reduced cytotoxic T-cell infiltration [73].

Clinical trials reflect these biological barriers. Nivolumab monotherapy demonstrated only modest activity in platinum-resistant OC, and the phase III NINJA trial failed to improve progression-free or overall survival compared with chemotherapy [74,75]. Similarly, pembrolizumab and avelumab have shown ORRs of only 8–10%, while combinations with chemotherapy or antiangiogenic agents have not consistently improved outcomes [72]. Overall, OC resistance to ICI is driven by an immunosuppressive TME, low neoantigen load, impaired antigen presentation, and immune exclusion. These mechanisms help explain the consistently low response rates observed in clinical trials of ICI monotherapy in ovarian cancer.

#### 4.2.9. Why Clear Cell Ovarian Carcinoma Responds Better to Immune Checkpoint Inhibition

Ovarian clear cell carcinoma (OCCC) is a distinct ovarian cancer subtype with molecular and immunological features associated with greater sensitivity to immune checkpoint inhibition. OCCC accounts for approximately 25% of ovarian cancers in Asian populations but less than 10% in Western populations. A defining feature of OCCC is the high prevalence of ARID1A mutations, occurring in approximately 50–60% of cases [76]. ARID1A deficiency impairs DNA repair, increases genomic instability and neoantigen burden, and thereby enhances tumor immunogenicity [70].

Compared with other ovarian cancer subtypes, OCCC frequently demonstrates increased PD-L1 expression and greater infiltration by CD8^+^ tumor-infiltrating lymphocytes, particularly in ARID1A-mutated tumors [76]. Clinical studies have reported response rates of 11–25% in OCCC patients treated with ICIs, exceeding the rates observed in unselected ovarian cancer populations. Combination strategies involving ICIs and antiangiogenic agents have shown ORRs up to 38.5% in selected cohorts, although responses remain heterogeneous [72].

The biological rationale for improved ICI responsiveness in OCCC also relates to the association between ARID1A deficiency and mismatch repair deficiency (dMMR), a recognized predictor of ICI sensitivity. ARID1A-deficient tumors additionally express elevated levels of immune checkpoints, including PD-1, PD-L1, LAG3, and TIM3, suggesting a pre-existing but exhausted immune response that may be restored through checkpoint blockade [76]. In contrast to HGSOC, which is typically characterized by a highly suppressive tumor microenvironment, OCCC more frequently exhibits increased PD-L1 expression, CD8^+^ T-cell infiltration, and ARID1A-associated immunogenicity, potentially contributing to improved responsiveness to immune checkpoint inhibition [77].

#### 4.2.10. Biological Rationale for Combination Strategies

The limited efficacy of ICI monotherapy in ovarian cancer has prompted investigation of combination strategies designed to overcome immune resistance within the tumor microenvironment. Chemotherapy is a major partner because it can induce immunogenic cell death, enhance tumor-antigen release, and promote dendritic-cell activation and T-cell priming. Neoadjuvant chemotherapy has also been shown to increase stromal TILs and PD-L1 expression, suggesting induction of a more inflamed microenvironment [71]. However, trials such as JAVELIN 200 combining chemotherapy with ICIs have not consistently improved survival outcomes [72].

Antiangiogenic therapy is another promising approach because VEGF contributes to immune suppression by impairing dendritic-cell maturation, recruiting Tregs and MDSCs, and restricting immune-cell trafficking [71]. VEGF inhibition with bevacizumab may normalize tumor vasculature and improve immune infiltration, particularly in immune-excluded tumors. Early studies combining anti-VEGF therapy with ICIs have shown encouraging activity, especially in platinum-sensitive disease [72].

Strong rationale also supports combining PARP inhibitors (PARPi) with ICIs. PARPi induces DNA damage and activates the STING pathway, increasing type I interferon signaling and antigen presentation [70]. At the same time, PARPi upregulate PD-L1 expression through ATM-ATR-CHK1 signaling, providing a mechanism of adaptive immune resistance that may be overcome with checkpoint blockade. Clinical trials including MEDIOLA, TOPACIO, and ATHENA have demonstrated encouraging responses with PARPi-ICI combinations in both BRCA-mutated and BRCA wild-type ovarian cancers [72].

Additional strategies include dual checkpoint blockade targeting PD-1 and CTLA-4, as well as therapies directed against alternative checkpoints such as LAG3 and TIM3 [71]. Vaccines, dendritic-cell therapies, bispecific T-cell engagers, and adoptive cell therapies, including TIL and CAR T-cell approaches, are also under investigation, particularly in combination with ICIs [72]. Recent multi-omic studies further show that HRD-associated tumors display inflammatory immune phenotypes, whereas FBI-driven tumors exhibit immune exclusion and elevated TGF-β signaling, emphasizing the need for biomarker-driven combination strategies [73].

Importantly, not all combination strategies provide equivalent clinical benefit, which may reflect differences in their ability to modulate the tumor immune microenvironment. Anti-angiogenic agents such as lenvatinib may enhance immunotherapy efficacy by normalizing tumor vasculature, improving T-cell infiltration, and reducing immunosuppressive cell populations, thereby creating a microenvironment more conducive to antitumor immune responses. In contrast, combinations lacking a clear mechanistic synergy, such as empiric pairing with chemotherapy or PARP inhibitors in unselected populations, may fail to overcome dominant resistance mechanisms. These differences may explain why certain combinations demonstrate more consistent clinical activity than others.

## 5. Conclusions

Although ICIs have produced durable clinical benefit across multiple malignancies, their impact in OC has thus far remained limited. Data from large randomized trials consistently demonstrate that PD-1/PD-L1 blockade, whether administered as monotherapy or integrated into first-line chemotherapy and maintenance strategies, does not confer a meaningful survival advantage in biomarker-unselected populations. These findings reflect the complex and frequently immunosuppressive immune microenvironment of OC, which is characterized by variable antigenicity, impaired antigen presentation, limited effector T-cell infiltration, and enrichment of regulatory immune subsets.

Nevertheless, selected contexts of immunotherapeutic vulnerability have emerged. Dual checkpoint blockade targeting PD-1 and CTLA-4 yields higher response rates than PD-1 inhibition alone, particularly in CCOC and other molecularly distinct subgroups, supporting the concept that histologic and genomic features shape immunologic sensitivity. Combination strategies incorporating ICI with anti-angiogenic agents, PARP inhibitors, or cytotoxic chemotherapy further suggest that modulation of tumor vasculature, DNA damage response, and immunogenic cell death may partially overcome intrinsic resistance. In parallel, investigation of alternative inhibitory pathways, including TIM-3, highlights the importance of addressing adaptive immune escape mechanisms, although clinical activity remains early and requires further validation.

Future clinical trials should adopt more biologically informed and testable designs. First, patient selection should be prospectively stratified based on integrated biomarkers, including histologic subtype, PD-L1 expression, and immune microenvironment characteristics, rather than enrolling unselected populations. Second, combination strategies should be developed based on a clear mechanistic rationale, particularly those targeting angiogenesis-driven immune suppression or T-cell exclusion. Third, early-phase trials should incorporate translational endpoints, such as changes in immune cell infiltration or checkpoint expression, to better define mechanisms of response and resistance.

## Figures and Tables

**Table 1:** Key phase II clinical trials of PD-1–based immune checkpoint inhibition in ovarian cancer.

Trial (Phase)	Regimen/Target	Population	Key Efficacy Outcomes	Key Safety Outcomes
PEACOCC (Phase II)	Pembrolizumab (anti–PD-1)	Clear cell ovarian carcinoma (CCOC), multiple prior lines	PFS-12 weeks: 42%|Median PFS: 2.7 mo|ORR: 25% Median OS: 14.8 mo	Grade 3 TRAEs: 19%|Grade 4–5 TRAEs: 0%
NCT05296512 (Phase II)	Pembrolizumab + lenvatinib (anti–PD-1 + anti-angiogenic TKI)	Recurrent/persistent CCOC	6-mo PFS: ~76% Median PFS: 10.9 mo|ORR: 30%	Most common any-grade TRAEs: hypertension 71%, hypothyroidism 66%, fatigue 60%; detailed grade ≥3 rates NR
KEYNOTE-100 (Phase II)	Pembrolizumab (anti–PD-1)	Recurrent advanced OC (heavily pretreated; 2 cohorts)	Median PFS: ~2.1 mo|ORR: 8.0% (cohort A), 9.9% (cohort B); higher ORR in CPS ≥10	Any-grade TRAEs: 61.9%|Grade 3–4 TRAEs: 23.8%|Treatment-related deaths: NR
MoST-CIRCUIT (Phase II; NCT04969887)	Nivolumab + ipilimumab (anti–PD-1 + anti–CTLA-4)	Advanced CCOC + clear-cell endometrial carcinoma (CCEC), ≤1 prior line	ORR: 54% (12% CR, 42% PR)|ORR in ovarian CCOC cohort: 55%|6-mo PFS: 58%|Median PFS/OS: NR (not reached)	Grade 3–4 irAEs: ~35%|Grade 5 TRAEs: 1 (immune myocarditis)

Abbreviations: CCOC, clear-cell ovarian carcinoma; CCEC, clear-cell endometrial carcinoma; OC, ovarian cancer; ORR, objective response rate; CR, complete response; PR, partial response; OS, overall survival; PFS, progression-free survival; mo, months; TKI, tyrosine kinase inhibitor; PD-1, programmed cell death protein 1; CTLA-4, cytotoxic T-lymphocyte-associated protein 4; TRAEs, treatment-related adverse events; irAEs, immune-related adverse events; NR, not reported.

**Table 2:** Key phase III trials of PD-1/PD-L1 immune checkpoint inhibition strategies in ovarian cancer.

Trial (Phase)	Regimen/Strategy	Population/Setting	Key Efficacy Outcomes (PFS/OS)	Key Safety Outcomes
ATHENA-COMBO (Phase III)	Rucaparib + nivolumab vs. rucaparib	First-line maintenance after response to platinum chemotherapy	Median PFS: 15.0 mo (combo) vs. 20.2 mo (rucaparib)	Grade ≥3: anemia 27.1% vs. 28.6%, neutropenia 25.4% vs. 15.4%, ALT/AST increase 21.2% vs. 10.0%
FIRST / ENGOT-OV44 (Phase III)	Dostarlimab + chemotherapy → maintenance niraparib + dostarlimab (±bevacizumab) vs. control	Newly diagnosed advanced OC	Median PFS: 20.6 mo vs. 19.2 mo; OS: no significant difference (immature)	Any-grade and grade ≥3 toxicity NR; “no new safety signals” reported
KEYNOTE-B96/ENGOT-ov65 (Phase III)	Pembrolizumab + weekly paclitaxel (±bevacizumab) vs. placebo	Platinum-resistant recurrent OC (PROC)	PFS improved (overall and PD-L1^+^ subgroup); OS improved in PD-L1^+^ subgroup (phase III, published data)	Grade ≥3 toxicity NR (topline available only)
JAVELIN Ovarian 200 (Phase III)	Avelumab vs. PLD vs. avelumab + PLD	Platinum-resistant OC	Median PFS: 1.9 mo (avelumab) vs. 3.7 mo (combo) vs. 3.5 mo (PLD)|Median OS: 11.8 vs. 15.7 vs. 13.1 mo	Serious TRAEs: 18% (combo) vs. 11% (PLD) vs. 7% (avelumab); grade 5 TRAEs reported (PLD, avelumab)
IMagyn050 (Phase III)	Atezolizumab + chemotherapy + bevacizumab vs. placebo	Newly diagnosed advanced OC	Median PFS: 19.5 mo (atezo) vs. 18.4 mo (control); OS not improved	Grade 3–4 neutropenia: 21% vs. 21%; hypertension: 18% vs. 20%; anemia: 12% vs. 12%
JAVELIN Ovarian 100 (Phase III)	Carboplatin/paclitaxel ± avelumab (concurrent and/or maintenance)	Treatment-naive stage III–IV EOC	Futility met; median PFS: 16.8 mo (avelumab maintenance) vs. 18.1 mo (avelumab concurrent+maint); control: NR/NE.	Serious AEs: 28% vs. 36% vs. 19% (avelumab maint vs. combo vs. control); grade 5 <1% in avelumab arms

Abbreviations: AE, adverse event; atezo, atezolizumab; ALT, alanine aminotransferase; AST, aspartate aminotransferase; EOC, epithelial ovarian cancer; NE, not estimable; NR, not reported; OC, ovarian cancer; OS, overall survival; PFS, progression-free survival; PD-L1, programmed death-ligand 1; PD-L1^+^, PD-L1 positive; PLD, pegylated liposomal doxorubicin; PROC, platinum-resistant ovarian cancer; TRAEs, treatment-related adverse events.

**Table 3:** Key trials of CTLA-4–based checkpoint blockade (alone or in combination) in ovarian cancer.

Trial (Phase)	Regimen/Target	Population/Setting	Key Efficacy Outcomes	Key Safety Outcomes
NRG-GY003 (Phase II)	Nivolumab vs. nivolumab + ipilimumab (PD-1 ± CTLA-4)	Recurrent/persistent epithelial OC/PPC/FTC; platinum-free interval <12 mo; 1–3 prior regimens	Median PFS: 3.9 mo (combo) vs. 2.0 mo (nivo)|ORR at 6 mo: 31.4% vs. 12.2%	Grade ≥3 TRAEs: 49% (combo) vs. 33% (nivo) Grade 5: 0%
BrUOG 354 (Phase II)	Nivolumab vs. nivolumab + ipilimumab	Previously treated clear-cell gynecologic carcinomas (ovarian CCOC + endometrial CCC)	Median PFS: 5.6 mo (combo) vs. 2.2 mo (mono)|6-mo PFS: 43.8% vs. 19.1% Median OS: 24.7 vs. 17.3 mo	Serious TRAEs: 47% (combo) vs. 21% (mono); detailed grade ≥3 NR
NCT03026062 (Phase II)	Tremelimumab +/− durvalumab	Platinum-resistant HGSOC, heavily pretreated	Median PFS: no meaningful difference between sequential vs. combination	Safety: NS/NR
KGOG-3046/TRU-D (Phase II)	Neoadjuvant chemotherapy + durvalumab + tremelimumab	Stage IIIC–IVB newly diagnosed EOC receiving NACT + interval debulking surgery	12-mo PFS: 63.6%|24-mo PFS: 45%|30-mo PFS: ~40%	Grade ≥3 toxicity reported (~30–35%); treatment-related death: no
NCT02571725 (Phase II)	Tremelimumab ± olaparib (CTLA-4 ± PARP inhibition)	Recurrent platinum-sensitive OC	Clinical benefit rate ~46%; mature PFS/OS NR	Grade 3 rash 13%; grade 3 hepatitis 8%; grade 3 colitis 8%; grade ≥4: 0%; treatment-related deaths: 0

Abbreviations: CCC, clear-cell carcinoma; CCOC, clear-cell ovarian carcinoma; EOC, epithelial ovarian cancer; FTC, fallopian tube cancer; HGSOC, high-grade serous ovarian cancer; NACT, neoadjuvant chemotherapy; NR, not reported; NS, not significant; OC, ovarian cancer; ORR, objective response rate; OS, overall survival; PARP, poly(ADP-ribose) polymerase; PD-1, programmed cell death protein 1; CTLA-4, cytotoxic T-lymphocyte-associated protein 4; PPC, primary peritoneal cancer; PFS, progression-free survival; mo, months; TRAEs, treatment-related adverse events.

**Table 4:** Clinical development of TIM-3–targeting immune checkpoint strategies (ovarian-cancer-eligible populations).

Agent/Trial (Phase)	Regimen/Target	Population/Setting	Key Efficacy Outcomes	Key Safety Outcomes
Lomvastomig (RO7121661) (Phase I; NCT03708328)	PD-1×TIM-3 bispecific antibody	Advanced/metastatic solid tumors (OC eligible)	Preliminary; no ovarian-specific outcomes reported; efficacy immature	Safety: NS/NR
INCAGN02390 (Phase I/II)	Anti–TIM-3 monoclonal antibody	Advanced/metastatic solid tumors (often heavily pretreated); OC eligible	Median PFS: 1.9 mo; OS immature; PR: 2.5%; disease control rate 18%	TRAEs overall NR; grade ≥3 in <8% reported; selected grade 3: rash 13%, hepatitis 8%, colitis 8%; grade ≥4: 0%
LY3415244 (Phase I)	TIM-3×PD-L1 bispecific antibody	Advanced solid tumors (OC eligible)	Trial terminated early due to immunogenicity; minimal efficacy signal	High immunogenicity; anaphylactic infusion reactions reported; treatment-related deaths: 0%

Abbreviations: OC, ovarian cancer; OS, overall survival; PD-1, programmed death 1; PD-L1, programmed death ligand 1; TIM-3, T-cell immunoglobulin and mucin-domain containing-3; PR, partial response; PFS, progression-free survival; mo, months; NR, not reported; NS, not significant/not stated; TRAEs, treatment-related adverse events.

**Table 5:** Cross-strategy comparison of immune checkpoint inhibition approaches in ovarian cancer.

Strategy	Representative Trials	Typical Efficacy Signal	Typical Grade ≥3 Toxicity
PD-1/PD-L1 monotherapy	PEACOCC; KEYNOTE-100; JAVELIN Ovarian 200 (mono)	Median PFS~1.9–2.7 mo; ORR generally low (higher in selected subtypes)	~19–24%
PD-1 + anti-angiogenic therapy	Pembrolizumab + lenvatinib (NCT05296512)	Median PFS~10.9 mo (CCOC); 6-mo PFS~76%; ORR~30%	NR (frequent HTN, fatigue, hypothyroidism reported)
PD-1 + CTLA-4 dual checkpoint blockade	NRG-GY003; BrUOG 354; MoST-CIRCUIT; TRU-D	Median PFS~3.9–5.6 mo; higher ORR (esp. clear cell)	~35–50%
CTLA-4 + PD-L1 (non-classical dual ICI)	NCT03026062	No clear PFS benefit	NR
CTLA-4 + PARP inhibitor	NCT02571725	CBR reported: mature PFS/OS NR	Grade 3 toxicities reported; grade ≥4: 0%
TIM-3 monotherapy	INCAGN02390	Median PFS~1.9 mo; limited activity	<8% (reported)
TIM-3 + PD-1/PD-L1 (bispecifics)	Lomvastomig; LY3415244	NR (no mature ovarian-specific efficacy)	Variable; LY3415244: immunogenicity risk

Abbreviations: CBR, clinical benefit rate; CCOC, clear-cell ovarian carcinoma; CTLA-4, cytotoxic T-lymphocyte-associated protein 4; HTN, hypertension; ICI, immune checkpoint inhibitor; mo, months; NR, not reported; ORR, objective response rate; OS, overall survival; PARP, poly(ADP-ribose) polymerase; PD-1, programmed cell death protein 1; PD-L1, programmed death-ligand 1; PFS, progression-free survival; TIM-3, T-cell immunoglobulin and mucin-domain containing-3.

**Table 6:** Biomarkers of response and resistance to immune checkpoint inhibition in ovarian cancer.

Biomarker	Biological Rationale	Prevalence in OC	Predictive Value for ICI Response	Clinical Utility
PD-L1 expression (CPS/TPS)	Inhibitory ligand suppressing T-cell activity via PD-1	~40%	Weak positive association; many PD-L1^+^ tumors do not respond	Not sufficient alone
MSI-H/dMMR	High neoantigen load from defective DNA mismatch repair	~3.9%	Strong predictor in MSI-H tumors	Useful to identify rare candidates
HRD/BRCA mutation	Genomic instability, increased immune signaling	~50% HRD in HGSOC	Not predictive of ICI monotherapy	Relevant mainly for combinations
Clear cell histology	ARID1A mutation, higher PD-L1, higher TILs	~5–10%	Consistently higher response rates	Most robust clinical marker
CD8^+^ TIL density	Pre-existing antitumor immunity	Variable	Strong positive predictor	High
Exhausted CD8^+^PD-1^+^ T cells	Targetable dysfunctional effector pool	Subset-dependent	Positive indicator	Emerging
VEGF/angiogenic signature	Immune exclusion, T-cell suppression	Common	Predicts resistance to ICI alone	Supports anti-VEGF combinations

Abbreviations: ARID1A, AT-rich interactive domain-containing protein 1A; BRCA, Breast cancer susceptibility gene; CD8^+^, Cluster of differentiation 8 positive; CPS, Combined Positive Score; dMMR, Deficient mismatch repair; HGSOC, High-grade serous ovarian cancer; HRD, Homologous recombination deficiency; ICI, Immune checkpoint inhibitor; MSI-H, Microsatellite instability–high; OC, Ovarian cancer; PD-1, Programmed cell death protein 1; PD-L1, Programmed death-ligand 1; TILs, Tumor-infiltrating lymphocytes; TPS, Tumor proportion score; VEGF, Vascular endothelial growth factor.

## Data Availability

No new data were created or analyzed in this study. Data sharing is not applicable to this article.

## List of Abbreviations Used in the Text

AE—adverse event; ALT—alanine aminotransferase; APC—antigen-presenting cell; ARID1A—AT-rich interactive domain-containing protein 1A; AST—aspartate aminotransferase; B7-DC—B7 dendritic cell ligand; B7-H1—B7 homolog 1; BRCA1—breast-cancer susceptibility gene 1; BRCA2—breast-cancer susceptibility gene 2; CAR—chimeric antigen receptor; CAR-NK—chimeric antigen receptor natural killer cell; CAR-T—chimeric antigen receptor T cell; CBR—clinical benefit rate; CCEC—clear-cell endometrial carcinoma; CCOC—clear-cell ovarian carcinoma; COX-2—cyclooxygenase-2; CPS—combined positive score; CR—complete response; CTLA-4—cytotoxic T-lymphocyte–associated protein 4; DC—dendritic cell; dMMR—deficient mismatch repair; EOC—epithelial ovarian cancer; FIGO—International Federation of Gynecology and Obstetrics; FTC—fallopian tube cancer; Gal-9—galectin-9; HGSC—high-grade serous carcinoma; HGSOC—high-grade serous ovarian cancer; ICI—immune checkpoint inhibitor; irAE—immune-related adverse event; LGSC—low-grade serous carcinoma; MHC—major histocompatibility complex; MSI—microsatellite instability; MSI-H—microsatellite instability, high; NE—Not estimable; NK cell—natural killer cell; NR—not reported; OC—ovarian cancer; ORR—objective response rate; OS—overall survival; PARP—poly(ADP-ribose) polymerase; PD-1—programmed-cell-death protein 1; PD-L1—programmed death-ligand 1; PD-L2—programmed death-ligand 2; PFS—progression-free survival; PLD—pegylated liposomal doxorubicin; PR—partial response; PROC—platinum-resistant ovarian cancer; RECIST—Response Evaluation Criteria in Solid Tumors; TCR—T-cell receptor; TERT—telomerase reverse transcriptase; TIL—tumor-infiltrating lymphocyte; TIM-3—T-cell immunoglobulin and mucin-domain-containing-3; TKI—tyrosine kinase inhibitor; TPS—tumor proportion score; TRAEs—treatment-related adverse events; Treg—regulatory T cell; VEGF—vascular endothelial growth factor.
